# Performance of 16S Metagenomic Profiling in Formalin-Fixed Paraffin-Embedded versus Fresh-Frozen Colorectal Cancer Tissues

**DOI:** 10.3390/cancers13215421

**Published:** 2021-10-29

**Authors:** Alessandra Borgognone, Garazi Serna, Marc Noguera-Julian, Lidia Alonso, Mariona Parera, Francesc Català-Moll, Lidia Sanchez, Roberta Fasani, Roger Paredes, Paolo Nuciforo

**Affiliations:** 1 IrsiCaixa AIDS Research Institute, Hospital Universitari Germans Trias i Pujol, 08916 Badalona, Barcelona, Spain; aborgognone@irsicaixa.es (A.B.); mnoguera@irsicaixa.es (M.N.-J.); mparera@irsicaixa.es (M.P.); fcatala@irsicaixa.es (F.C.-M.); 2 Vall d’Hebron Institute of Oncology (VHIO), Vall d’Hebron University Hospital, 08035 Barcelona, Spain; gserna@vhio.net (G.S.); lalonso@vhio.net (L.A.); lsanchez@vhio.net (L.S.); rfasani@vhio.net (R.F.); 3 Faculty of Medicine, University of Vic–Central University of Catalonia (UVic–UCC), 08500 Vic, Barcelona, Spain; 4 Facultat de Medicina, Universitat Autonoma de Barcelona (UAB), 08193 Barcelona, Spain; 5 Fight AIDS Foundation, Infectious Diseases Department, Hospital Universitari Germans Trias i Pujol, 08916 Badalona, Barcelona, Spain; 6 Infectious Diseases Service, Hospital Universitari Germans Trias i Pujol, 08916 Badalona, Barcelona, Spain; 7 Center for Global Health and Diseases, Department of Pathology, Case Western Reserve University, Cleveland, OH 44106, USA

**Keywords:** microbiome, colorectal cancer, 16S gene sequencing, RNA in situ hybridization, FFPE

## Abstract

**Simple Summary:**

The analysis of colorectal cancer (CRC) gut microbiota can reveal crucial aspects of carcinogenesis and variation of treatment responses. Formalin-fixed, paraffin-embedded (FFPE) tissues represent an invaluable resource for studies in cancer genomics; however, their use in high-throughput metagenomic studies has been questioned due to several limitations in the DNA quality. In this study, we evaluated the impact of sample preservation on CRC-associated microbiota characterization. Using 16S rRNA sequencing and RNA in situ hybridization (RNA-ISH), we found differences in the comparison between paired FFPE and fresh frozen (FF) tissues, mostly derived from contamination issues. A quality index was also outlined to potentially assess the reliability of microbiome profiling obtained from FFPE DNA samples. These results suggest that tissular CRC microbiome studies should preserve internal coherence by using either FFPE or FF samples but not necessarily both.

**Abstract:**

Formalin-fixed, paraffin-embedded (FFPE) tissues represent the most widely available clinical material to study colorectal cancer (CRC). However, the accuracy and clinical validity of FFPE microbiome profiling in CRC is uncertain. Here, we compared the microbial composition of 10 paired fresh-frozen (FF) and FFPE CRC tissues using 16S rRNA sequencing and RNA-ISH. Both sample types showed different microbial diversity and composition. FF samples were enriched in archaea and representative CRC-associated bacteria, such as *Firmicutes*, *Bacteroidetes* and *Fusobacteria*. Conversely, FFPE samples were mainly enriched in typical contaminants, such as *Sphingomonadales* and *Rhodobacterales*. RNA-ISH in FFPE tissues confirmed the presence of CRC-associated bacteria, such as *Fusobacterium* and *Bacteroides*, as well as *Propionibacterium* allowing discrimination between tumor-associated and contaminant taxa. An internal quality index showed that the degree of similarity within sample pairs inversely correlated with the dominance of contaminant taxa. Given the importance of FFPE specimens for larger studies in human cancer genomics, our findings may provide useful indications on potential confounding factors to consider for accurate and reproducible metagenomics analyses.

## 1. Introduction

Increasing advances in high-throughput sequencing technologies have provided remarkable insights into the role played by the human microbiome in the host’s health status and pathological conditions [1], including colorectal cancer (CRC) [2]. Comparative metagenomics analyses on fecal and mucosal samples have explored the gut microbiota of individuals with CRC, resulting in the identification of bacterial groups that have a critical role in oncogenesis and tumor progression [3]. In particular, increased abundance of the gut pathogenic bacterium *Fusobacterium nucleatum* in CRC patients correlated with shorter survival [4], resistance to chemotherapy [5] and molecular alterations [6]. Several mechanistic studies have hypothesized that the close interaction of diverse microbial communities with host intestinal cells and immune system may induce alterations in the metabolic environment, thus directly or indirectly influencing mutagenesis rate and tumor progression [7]. As a result, to deepen understanding in the field of tumor-associated microbiome, the demand for larger cohorts of patient samples has dramatically increased. In recent years, stool material has emerged as the most common biospecimen used to characterize the human gut microbiota because of the non-invasive nature of its retrieval and large amount of biomass. However, stool-derived profiles are generally more representative of microbial communities present in the intestinal lumen rather than mucosa-associated microbiota adherent to the host tissue and may be less sensitive to localized changes in the surface of the colorectal wall [8]. Hence, these limitations have fueled ongoing research into tissue biopsies that would more accurately reflect local mucosal communities [9].

At present, fresh frozen (FF)-resected tissues are considered the “gold standard” for sequencing-based microbiome studies due to several advantages in preserving the DNA (i.e., immediate freezing, less fragmentation, limited handling and lower contamination or storage-derived issues). However, the frozen material is not collected as part of clinical routine and its use is generally limited to prospective or cross-sectional analyses [10].

To overcome these drawbacks, the use of formalin-fixed paraffin-embedded specimens (FFPE) has been explored [11,12]. Compared with the frozen material, FFPE tissues are more suitable for relatively simple long-term storage at room temperature and are widely available from biobanks in pathology departments [13]. Although this biotype harbors a great potential for expanding metagenomics studies (i.e., allowing access to clinical samples from a wide range of locations and times), FFPE specimens carry several limitations for genomic analysis [14] mostly derived from the formalin fixation process and storage that negatively impact the DNA integrity (e.g., cross-linking, fragmentation, and mutations) [15]. In this regard, only a limited number of comparative studies have investigated the potential to extract reliable information from both FF and FFPE specimens. For instance, previous reports successfully used FFPE specimens to characterize the microbiota of pre-term infants with necrotizing enterocolitis using 16S rRNA sequencing [16] or genomic alterations in colon and breast cancers by exome capture sequencing [17]. By contrast, 16S rRNA sequencing analyses of non-neoplastic gastric tissues [18] and brain specimens in a cohort of Alzheimer’s patients [19] showed that the microbial community profiled in FFPE tissues did not fully recapitulate that of their paired FF tissues. Recently, intratumor bacteria have been successfully characterized across distinct cancer types in both FF and FFPE tissues, using a multiplexed 16S rRNA sequencing protocol [20]. Nevertheless, the impact of sample preservation on CRC-associated microbiota has not been fully elucidated.

To assess the feasibility of typing the CRC-associated microbiota from FFPE biospecimens, the current study compared CRC-related microbiota from paired FF and FFPE tissue samples using 16S rRNA sequencing. A special focus was drawn on the characterization of *F. nucleatum* in CRC specimens from both FF and FFPE sample types. Finally, high resolution in situ analyses in tumor samples was used to validate potential microbial biomarkers identified by amplicon sequencing.

## 2. Materials and Methods

### 2.1. Sample Collection

The study group comprised 10 non-consecutive patients who were diagnosed with colorectal cancer at Vall d’Hebron University Hospital between 2010 and 2014 and had available FF and FFPE tissue for analysis. Clinicopathologic data is shown in Supplementary Table S1. All tumor samples were collected at surgery from treatment-naïve patients. The study was approved by the Vall d’Hebron University Hospital institutional ethical review board.

### 2.2. DNA Extraction

Five curls of 10µm of each FF and FFPE samples were used for the DNA extraction. The minimum percentage of tumor cells was 10%, except for patient 134’s FF sample where the information was missing. DNA from FF samples was extracted using the DNeasy Blood&Tissue kit (50) (#69504, QIAGEN, Düsseldorf, Germany) following the manufacturer’s instructions.

DNA from FFPE samples was extracted using the Maxwell 16 FFPE Plus LEV DNA Purification kit (#AS1135, Promega Corporation, Madison, WI, USA) following the manufacturer’s instructions. Extracted DNA was then stored at −80 °C until sequencing.

### 2.3. Library Preparation for Illumina MiSeq Sequencing

The V3–V4 variable region from the 16S rRNA gene was amplified using the primer pair described in the MiSeq rRNA Amplicon Sequencing protocol developed by Illumina (San Diego, CA, USA), which included forward and reverse adapters (16S_F 5′-TCG TCG TCG GCA GCG TCA GAT GTG TAT AAG AGA CAG CCT ACG GGN GGC WGC AG-3′; 16S_R 5′-GTC TCG TGG GCT CGG AGA TGT GTA TAA GAG ACA GGA CTA CHV GGG TAT CTA ATC C-3′). Amplifications were performed in triplicate in 25 μL reaction volumes containing 12.5 μL of KAPA HiFi HotStart Ready Mix (KAPA HiFi HotStart DNA Polymerase, buffer, MgCl2, and dNTPs, KAPA Biosystems Inc., Wilmington, MA, USA), 5 μL of each primer at 1 μM and 2.5 μL of template DNA. Thermocycling parameters were as follows: initial denaturation step at 95 °C for 3 min, followed by 30 cycles of denaturation at 95 °C for 30 s, annealing at 55 °C for 30 s, extension at 72 °C for 30 s and a final extension step at 72 °C for 10 min. A PCR reaction with DNA-free water as the template (PCR no-template control) was loaded to assess for potential contamination. Following amplification, PCR products were run on a 1% agarose gel electrophoresis to confirm the expected amplicon size (~460 base pairs), triplicates were pooled together and stored at −30 °C until sequencing library preparation. Amplified DNA templates were purified for non-DNA molecules and Illumina sequencing adapters and dual indices were attached using Nextera XT index Kit (Illumina Inc.), followed by a corresponding PCR amplification program as described in MiSeq 16S rRNA Amplicon Sequencing protocol. After the second round of purification, amplicon libraries were quantified using a Quant-iTTM PicoGreen^®^ dsDNA Assay Kit (Invitrogen, Carlsbad, MA, USA) and diluted in equimolar concentrations (4 nM) for further pooling. Sequencing was performed on an Illumina MiSeqTM platform (Illumina Inc.) using the paired-end 300 base-length protocol at the genomics core facility in Germans Trias i Pujol research campus, in Badalona, Spain.

### 2.4. 16S rRNA Sequence Analysis

Sequencing outputs from the Illumina MiSeq platform were converted to fastq format and demultiplexed before downloading from Illumina BaseSpace Hub. The quality of raw reads was visualized using FastQC [21] and then the reads were imported into R (v3.5.2) [22] for analysis with the DADA2 package (v1.10.1) [23]. The pipeline was executed according to default parameters using maxEE = 4.10 in the filtering step. Briefly, reads were first filtered for quality (expected error per read ≤ 2), trimmed (10 nucleotides from the start of each read) and those with <350 base pairs after filtering and trimming were removed. After filtered read dereplication, consensus quality profiles were used in the denoising step to correct sequencing errors and generate amplicon sequence variants (ASVs). Chimeric sequences were subtracted using a consensus approach and paired-end reads merged together. Taxonomy was assigned by aligning high-quality reads to the Ribosomal Database Project (RDP) database [24] natively implemented in DADA2 and trained against the Greengenes reference database (v13.8) [25] and the resulting ASV table used for downstream analyses.

### 2.5. Statistical Analysis

R/phyloseq (v1.26.1) [26], vegan (v2.5-5) [27] ade4 (v1.7-13) [28] and ggplot2 (v3.2.0) [29] packages were used to estimate relative abundances, diversity measures and for data visualization. Specifically, relative abundances of taxa were based on ASV counts and normalization calculated as percentages (100 × (x/sum(x))). Alpha diversity (Shannon index) was determined using the R/phyloseq ‘estimate_richness’ function on rarefied ASV counts. Beta diversity measures were assessed using the Bray–Curtis and Jaccard distances and calculated based on normalized ASV counts. PERMANOVA (adonis) tests (R/vegan package) using Bray–Curtis and Jaccard distances were performed to test for potential associations between preservation method and microbiome composition. Differences in alpha diversity and relative abundances of taxa were evaluated using Wilcoxon signed-rank test for pairwise comparisons. For all statistical tests, *p*-values lower than 0.05 were considered significant. To identify discriminant bacterial signatures, the linear discriminant analysis effect size (LEfSe) algorithm [30] was applied using default recommended settings (α = 0.05 for pairwise Wilcoxon test and LDA score > 3). Spearman’s correlation coefficients and corresponding *p*-values adjusted for multiple comparisons by the Benjamini–Hochberg method were computed using ‘rcorr’ function within R package hmisc.

### 2.6. Species-Level Taxonomy Inference and Phylogenetic Tree Reconstruction

Representative ASVs were obtained using the ‘subset_taxa’ function (via R/phyloseq package) for discriminating putative species classification. Amplicon sequences (fasta format) were aligned to the NCBI Refseq [31] database using BLASTN [32] (max_target_seqs = 20), optimizing for highly similar sequences (MegaBLAST). The resulting hits were sorted first by e-value and ASVs were assigned a putative taxonomy with sequence identity ≥ 98% and coverage ≥ 90%. For *Fusobacterium*-associated ASVs, phylogenies were built as follows: a multiple alignment was computed with MUSCLE (v.3.8.31) [33]; the alignment was then trimmed with trimAl (v1.4) using the ‘gappyout’ option [34]; and the tree was built with IQ-TREE (v1.5.5) [35] with 1000 ultrafast bootstraps. The 16S rRNA gene reference sequences of *Fusobacterium* spp. for phylogenetic analysis were downloaded from Pathosystems Resource Integration Center (PATRIC) databases [36].

### 2.7. Microbial In Situ Hybridization (ISH) and Image Analysis

RNA in situ hybridization (RNA-ISH) was conducted using the RNAscope^®^ technology as described in Serna et al. 2020. The following ACD probes were used: B-Fusobacterium 23S RNA probe ACD (Cat nº 486411, accession no: CP003723), Propionibacterium acnes-16S RNA probe ACD (Cat nº 313939), Bacteroides-23S probe ACD (Cat nº 575449) and EB-16S-rRNA probe ACD (Cat nº 464469) for total bacteria analyses. RNA-ISH stained slides were digitized for signal quantification using a custom-made algorithm that automatically detected and counted individual and clustered red signals corresponding to bacteria mRNA molecules within a determined tumor region of interest (ROI) and within the total area. Results were expressed in counts. Samples with less than 100 counts of total bacteria were discarded as they failed to pass the quality control for RNA-ISH studies and ISH results from these samples were excluded from the analysis. For single bacteria analyses, samples with a minimum of 100 counts of the bacteria were considered positive for the study.

## 3. Results

### 3.1. Data Processing and Quality Control

A total of 21 samples were sequenced with Illumina MiSeq, including 10 paired FF and FFPE colorectal cancer biopsies and 1 negative PCR no-template control (NTC). Previous studies have reported the presence of bacterial DNA in tissue embedding media, such as paraffin [18,20]. Therefore, we initially included 10 paraffin controls (blank section obtained from the margins of each paraffin block) to evaluate the impact of the embedding process on microbiota profiling. After PCR amplification, V3–V4 amplicon bands were not detectable on the agarose gel electrophoresis in correspondence to blank paraffin controls (Figure S1). Given the extremely low microbial biomass, these samples were excluded for further processing.

Raw sequencing data comprised 1,324,033 paired-end reads generated from 21 samples (Table 1). All filtered samples had mean quality scores of over 35 and the resulting ASV table retained a median of 15,990 chimera-removed, high-quality reads per sample (range: 5808–36,337) (Table 1). Although raw reads were significantly higher in FFPE (*p* = 0.02, Figure S2A), no significant differences in filtered high-quality reads were found between FF and FFPE (paired Wilcoxon test, *p* = 0.85, Figure S2A). After examining filtered read counts, rarefaction curves of the ASV table indicated a saturation of diversity at 5000 reads/sample, except for sample 124-FF (Figure S2B).

**Figure 1:**
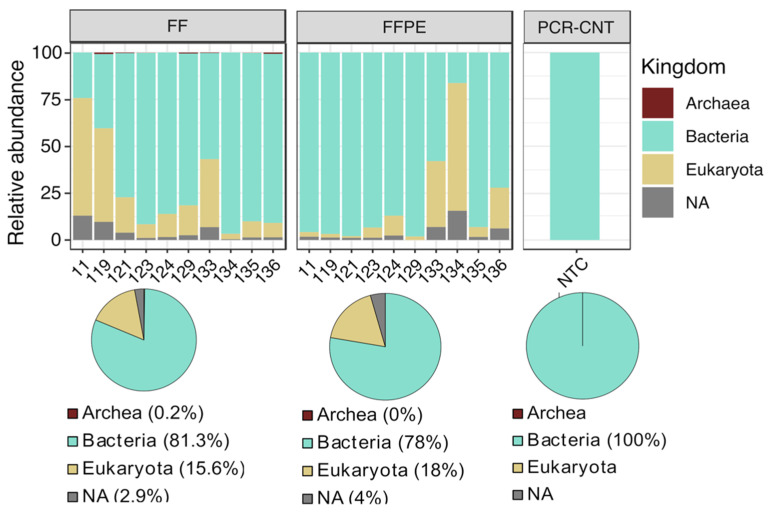
Taxa composition at the kingdom level. Bar plots showing global composition at the kingdom level. Individual samples are reported in the *x*-axis and relative abundance (as percentage) of taxa in the *y*-axis. Pie graphs below each panel display the relative abundance of identified taxa in the corresponding group. NA (not available) indicates unassigned ASVs at the kingdom taxonomy rank. Abbreviations: FF; fresh frozen, FFPE; formalin-fixed paraffin-embedded PCR-CNT; PCR no-template control and NTC; no-template control.

Both FF and FFPE groups were dominated by *Bacteria* (81.3% in FF vs. 78% in FFPE), followed by *Eukaryotes* (15.6 % in FF vs. 18% in FFPE) and ASVs without kingdom level assignment (2.9% in FF and 4% in FFPE), whereas *Archaea* were detected only in FF samples (0.2%) (Figure 1). ASVs matching with eukaryotic sequences were used to infer putative taxonomy, by using blastn vs. NCBI RefSeq databases. The query indicated that 99.4% of eukaryotic sequences matched with human sequences (23% corresponded to proteins broadly expressed in the gut) and other mammalian species (*Mus musculus* and *Pan troglodytes*), at lower proportions (0.6%). Therefore, eukaryotic sequences were filtered out from the ASV table. We found that the NTC had approximately 25,000 reads (Figure S2A and Table 1) and the composition was exclusively dominated by bacterial sequences (Figure 1). The presence of high levels of bacterial DNA in PCR no-template controls, mostly matching with general contaminants was also described in previous reports [20]. To assess for potential cross-contamination between samples, 339 ASVs associated with the NTC were taxonomically characterized at the genus scale. The microbial community in the NTC was mostly dominated by *Rhizobium* (56%), *Acinetobacter* (11%), *Lysinibacillus* (7%), *Delftia* (5%) and other taxa, described as general environmental contaminants [37] (Figure S3A). When we compared the two groups, these taxa were significantly more abundant in FFPE samples (*p* = 2 × 10^−5^), although at low proportions (Figure S3A,B). All the 339 NTC-associated ASVs were then in silico subtracted from the datasets. Furthermore, singletons were removed from the ASV table to filter out low-abundant sequences.

### 3.2. Comparison of the Microbial Diversity between FF and FFPE Tissues

**Figure 2:**
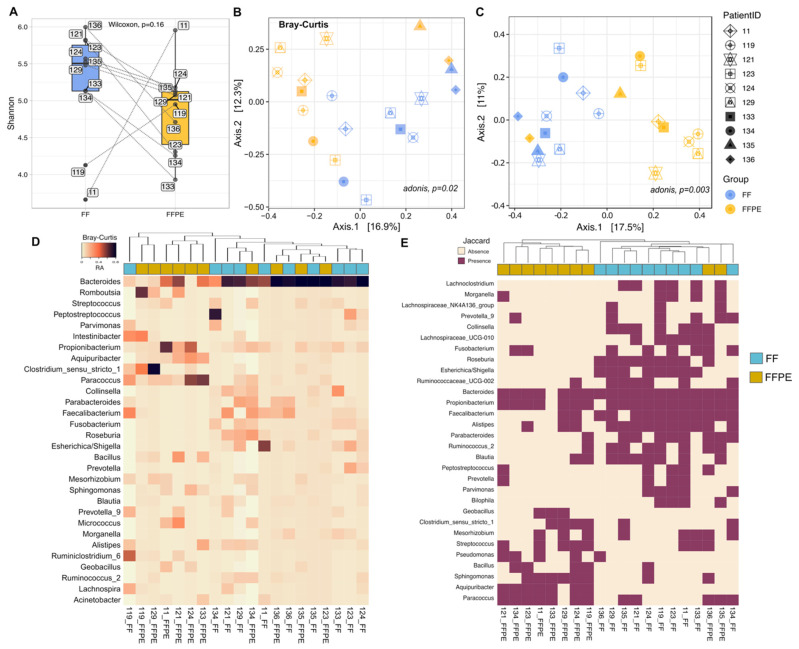
Alpha- and beta-diversity analysis of FF and FFPE tissue samples. (**A**) Box plots displaying Shannon diversity at the ASV level for each preservation method, with pairs connected by lines (*p* = 0.16, Wilcoxon test). (**B**,**C**) Principal coordinate analysis (PCoA) based on Bray–Curtis (**B**) and Jaccard (**C**) distances. Each point represents a unique sample. Sample pairs and preservation methods are differentiated by symbol shapes and colors, respectively. The proportion of variance corresponding to each principal component is reported in the corresponding axis. PERMANOVA-adonis r-squared and *p*-values are shown at the top of each plot. Both (**B**,**C**) share the same figure legend. (**D**,**E**) Heatmaps showing hierarchical clustering analysis of bacterial composition profiles based on Bray-Curtis (**D**) and Jaccard (**E**) distance metrics. The cluster analysis was generated using ASV abundances at the genus level (30 most abundant genera are reported) and Ward’s method (as linkage method). Colored bar beneath upper each heatmap indicates the preservation method (FF in blue and FFPE in yellow). Taxa are reported in row labels and sampleID indicated by column labels. Abbreviations: FF; fresh frozen and FFPE; formalin-fixed paraffin-embedded.

A general increase in alpha diversity (within-samples diversity) estimated by the Shannon Index was observed in FF (median = 5.5, IQR = 5.13–5.75) vs. FFPE (median = 5.01 IQR = 4.4–4.85), although differences were not statistically significant (Figure 2A).

In the beta diversity assessment, principal coordinate analysis (PCoA) based on both Bray–Curtis and Jaccard distances showed that the microbial community present in FFPE tended to cluster separately from the communities in FF samples, with the factor *PatientID* showing higher contribution to the variance (PERMANOVA, Bray–Curtis *p* = 0.002, r2 = 0.57 and Jaccard *p* = 0.003, r2 = 0.55) (Figure 2B,C). Hierarchical clustering based on Bray–Curtis distances displayed differences in the microbial abundance between the two groups, with some sample pairs clustering together (i.e., 119, 135 and 136) (Figure 2D). Six out of ten FFPE samples clustered together, with the most abundant genera including *Romboutsia*, *Propionibacterium, Aquipuribacter* and *Paracoccus.* In a separate cluster, mostly composed of FF samples, bacterial sequences were predominantly from *Collinsella, Faecalibacterium, Fusobacterium, Roseburia* and *Escherichia/Shigella*. Another cluster including samples from both groups was characterized by a Bacteroides-rich microbiota (Figure 2D). Hierarchical clustering based on the Jaccard distance showed a clear separation between FF and FFPE samples, with few exceptions (134-FF, 135-FFPE and 136-FFPE) (Figure 2E). The most prevalent bacterial groups in FFPE samples included *Paracoccus, Aquipuribacter, Sphingomonas, Streptococcus* and *Bacillus*, whereas microbiota profiles in FF samples were mainly distinguished by the presence of *Collinsella, Fusobacterium, Roseburia, Escherichia/Shigella, Faecalibacterium, Alistipes* and *Ruminococcus*. Yet, *Bacteroides* and *Propionibacterium* appeared to be ubiquitously distributed across both FF and FFPE samples.

### 3.3. Taxonomic Profiling and Discriminant Taxa between FF and FFPE Tissue Samples

**Figure 3:**
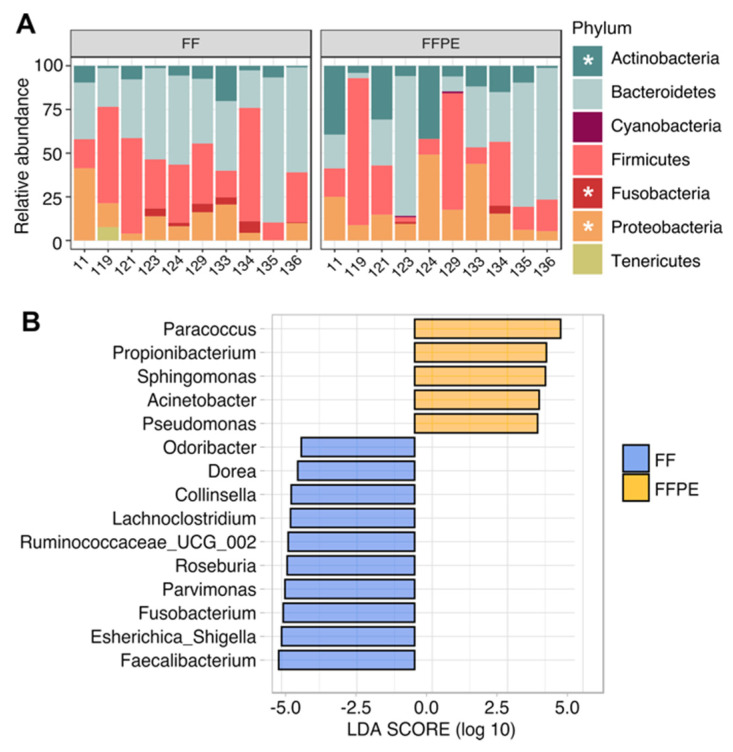
Discriminant taxa between FF and FFPE preservation methods. (**A**) Stacked bar chart showing bacterial phyla. Significantly different phyla between FF and FFPE are indicated by a white asterisk (*p* ≤ 0.05, Wilcoxon paired test). Individual samples are reported in the *x*-axis and relative abundance (as percentage) of taxa in the *y*-axis. (**B**) LEfSe rank plot of microbial differences between FF and FFPE tissue samples, expressed as LDA logarithmic score. Factorial pairwise Wilcoxon test with *p* ≤ 0.05 and LDA logarithmic score > 3 indicated statistical significance. Abbreviations: FF; fresh frozen, FFPE; formalin-fixed paraffin-embedded, NA, unidentified taxa and NTC, no-template control.

The taxonomic characterization indicated that one of the most remarkable differences between FF and FFPE sample pairs emerged from the archaeal content as reported in Figure 1. Specifically, archaeal sequences were detected exclusively in FF samples, accounting for up to 2% of the global composition (Figure S4A). Of these, 40% of archaeal-associated ASVs were assigned to *Crenarchaeota, Euryarchaeota* and *Woesearchaeota* phyla and detected in 5 out of 10 FF samples with relative abundances lower than 1% (Figure S4B). The bacterial composition was dominated by *Bacteroidetes* (43.3% in FF vs. 34.7% in FFPE), *Firmicutes* (34.1% in FF vs. 28.4% in FFPE), *Proteobacteria* (13.2% in FF vs. 19.6% in FFPE) and *Actinobacteria* (6.3% in FF vs. 19.6% in FFPE) in both groups (Figure 3A). Compared with their matched FF samples, the microbiota of FFPE tissues was significantly enriched in *Actinobacteria* (*p* = 0.002) and *Proteobacteria* (*p* = 0.008), and depleted in *Fusobacteria* (*p* = 0.036). Such differences were more evident at the order level, with *Enterobacteriales* (*p* = 0.009), *Coriobacteriales* (*p* = 0.014), *Fusobacteriales* (*p* = 0.036) and *Clostridiales* (*p* = 0.049) significantly increased in FF, whereas *Rhodobacterales* (*p* = 0.009), *Propionibacteriales* (*p* = 0.011), *Bacillales* (*p* = 0.022), *Sphingomonadales* (*p =* 0.022) and *Lactobacillales* (*p =* 0.032) significantly higher in FFPE samples (Figure S5). Discriminant analysis at the genus level showed concordance with differences observed at the order level (Figure 3B and Figure S5). *Paracoccus* (order Rhodobacterales), *Propionibacterium* (order Propionibacteriales) and *Sphingomonas* (order Sphingomonadales) were identified in FFPE, whereas *Collinsella* (order Coriobacteriales), *Dorea, Lachnoclostridium, Ruminococcaceae_UCG-002, Roseburia, Parvimonas* and *Faecalibacterium* (order Clostridiales), *Esherichica/Shigella* (order Enterobacteriales) and *Fusobacterium* (order Fusobacteriales) were detected in FF. Other genera found to be more abundant in FF tissues included *Odoribacter* (order Bacteroidales), while *Acinetobacter* and *Pseudomonas* (order Pseudomonadales) were found in FFPE samples (Figure 3B). Taken together, these results suggest that the preservation method has an impact on the microbiota composition of CRC tissues.

### 3.4. Putative Fusobacterium, Bacteroides and Propionibacterium Species Classification

Several multi-cohort studies have documented an enrichment of *Fusobacterium*, particularly *F. nucleatum*, in colorectal tumors [38]. In our dataset, sequences matching with *Fusobacterium* were detected in 6 FF and 2 FFPE samples (2.2% in FF vs. 0.6% in FFPE mean relative abundance, *p* = 0.036) (Figure S6A). Moreover, *Bacteroides* and *Propionibacterium*, both previously linked to the development of human gastric cancers [39], were detected in all tissue samples with few exceptions, although at different proportions (34.3% in FF vs. 29.6% in FFPE, *p* = 0.43 for *Bacteroides*; 2.5% in FF vs. 8.4% in FFPE global mean, *p* = 0.0092 for *Propionibacterium*) (Figure S6A). Analysis of published data from paired FF/FFPE non-tumoral gastric tissues [18] showed that *Propionibacterium* was higher in the FFPE group (21.8% in FF vs. 62.4% in FFPE, *p* = 0.062) (Figure S6B). As expected, *Fusobacterium* was present at very low abundance (0.33% in FF vs. 0.08% in FFPE, *p* = 0.18), whereas sequences from *Bacteroides* were not detected in this dataset (Figure S6B).

We then attempted to assign *Fusobacterium, Bacteroides* and *Propionibacterium* putative species by aligning corresponding ASVs to the 16S microbial database using BLASTN (see Methods). All *Propionibacterium* (n ASVs = 14) and *Bacteroides* (n ASVs =1) sequences were assigned to a single candidate species identified as *Cutibacterium acnes* strain JCM 6425 (formerly Propionibacterium acnes, 99.10% mean identity) and *Bacteroides dorei* strain 175 (99.57% mean identity), respectively (data not shown).

Sequence alignment generated from *Fusobacterium*-associated ASVs discriminated five putative species. *F. nucleatum* (99.29% mean identity) was the only species detected in FFPE (0.57% mean relative abundance) and the most abundant in FF group (1.78% mean relative abundance). Other putative species identified in FF tissue samples included *F. varium, F. mortiferum, F. simiae* and *F. periodonticum* (0.25%, 0.1%, 0.08% and 0.01% mean relative abundance, respectively), with 99.21% mean identity (Figure S7A). To validate the putative taxonomy assignment, the phylogenetic relationship between Fusobacterium sequences (ASVs) using IQ-TREE to build a maximum-likelihood tree (see Methods) was assessed. Individual 16S rRNA reference genes for *Fusobacterium* spp, along with that of *Fusobacterium*-associated ASVs were included to determine inter-, intra- and subspecies relationships. The phylogenetic analysis revealed two main branched clades of *Fusobacterium* phylotypes. *F. mortiferium* (28 ASVs + 16S reference gene) was phylogenetically close to *F. varium* (11 ASVs + 16S reference gene). Four detected *F. nucleatum* subspecies (49 ASVs + 16S reference gene) clustered in a different clade and were closely related to *F. simiae* (8 ASVs) and *F. periodonticum* (1 ASV + 16S reference gene) (Figure S7B). Considering that such phylogenetic patterns agreed with previous reports [40], it appears reasonable to suggest the validity of putative species classification herein described, also evidencing the predominance of *F. nucleatum* over other *Fusobacterium* species in FF samples from our dataset.

### 3.5. Comparison of CRC-Associated Bacteria Characterized by RNA-ISH and 16S rRNA Sequencing

RNA-ISH technique was performed on FFPE samples to validate in situ the results obtained by 16S rRNA sequence analysis. Results were expressed in counts. Samples with less than 100 counts of total bacteria (analyzed with the EB16S probe) quantified by image analysis were excluded from the analysis for not passing the quality control for RNA-ISH studies (Supplementary Table S2). To determine potential differences in the community structure profiled from the two methods, we used bacterial absence/presence (Table 2) summarized from quantitative data (Supplementary Table S2) for each sample pair. Bacterial presence was determined with the threshold of ≥100 and ≥10 bacterial counts for RNA-ISH and 16S rRNA sequencing, respectively.

**Figure 4:**
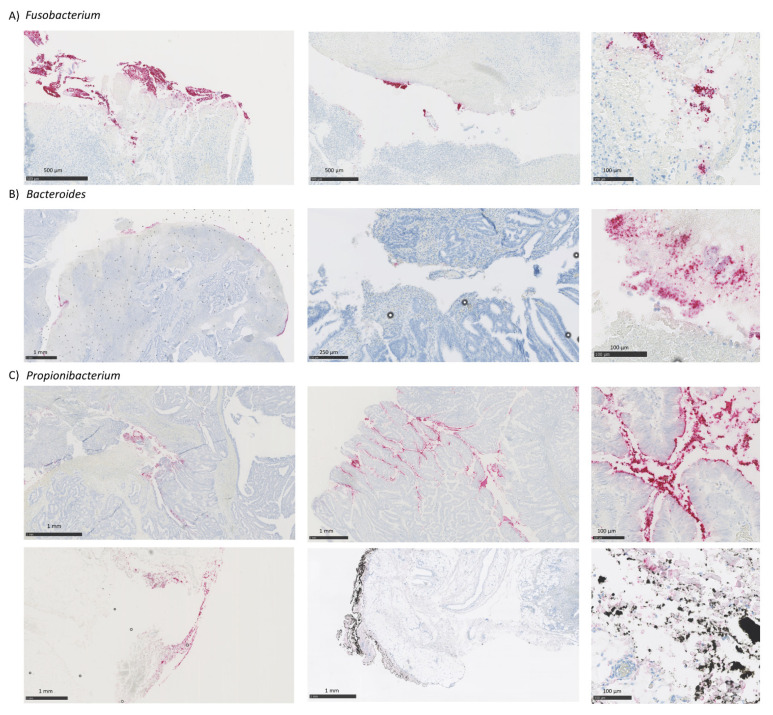
Representative RNA-ISH images in CRC tumor samples. (**A**) Representative images of *Fusobacterium* positive samples. (**B**) Representative images of *Bacteroides* positive samples. (**C**) Representative images of *Propionibacterium* positive samples showing the distinction between the tumor-associated *Propionibacterium* (top images) and the contaminant one (bottom images). Abbreviations: RNA-ISH; RNA in situ hybridization and CRC; colorectal cancer.

Concordance between methods for the detection of tumor-associated bacteria varied according to the bacteria analyzed. RNA-ISH confirmed the presence of tumor-associated *Fusobacterium* in all cases positive by 16S. Notably, *Fusobacterium* could be visualized by RNA-ISH also in FFPE samples that were negative by 16S but positive by 16S in FF samples (Figure 4, Table 2). When analyzing the *Propionibacterium*, RNA-ISH confirmed the presence of tumor-associated *Propionibacterium* in all FFPE samples as also shown by 16S analysis on the same sample type. There was one sample negative for 16S in FF that was positive in FFPE that could also be confirmed by RNA-ISH (Table 2). Moreover, RNA-ISH allowed the distinction between the tumor-associated *Propionibacterium* and the contaminant one, defining contaminant as the bacteria that was associated neither to tumor nor to adjacent normal mucosa, generally appearing in the outer margins of the samples (Figure 4). When analyzing the *Bacteroides*, RNA-ISH detected the presence of tumor-associated bacteria only in half (three out of six) of the positive FFPE samples by 16s, and three out of seven FF samples that were positive by 16S. (Figure 4, Table 2).

Bacterial content was also analyzed in adjacent normal mucosa by RNA-ISH. All bacteria were found significantly enriched in tumor tissue. *Propionibacterium* was visualized also in correspondence of adjacent non-tumoral mucosa while *Fusobacterium* and *Bacteroides* were tumor-associated (Figure S8A). A similar profile was observed in a published dataset of metagenomic analysis comparing FFPE normal mucosa and colorectal cancer tissues [9], with *Propionibacterium* sequences equally distributed in both groups and *Fusobacterium* and *Bacteroides* significantly increased in tumor tissues (Figure S8B).

### 3.6. Quality Assessment of FFPE DNA Based on 16S rRNA Amplicon Profiling

**Figure 5:**
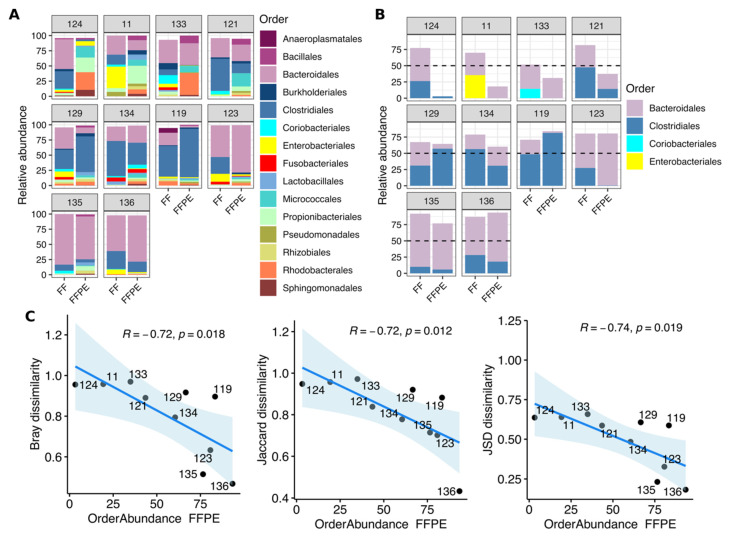
Correlation between community similarity and bacterial dominance in FF and FFPE sample pairs. (**A**) Comparison of bacterial composition (top 15 most abundant orders) between paired FF and FFPE samples. (**B**) Representation of two most abundant bacterial orders in FF tissues characterized in their matched FFPE samples. (**C**) Scatter plots showing spearman’s correlation between Bray–Curtis, Jaccard and Jensen–Shannon dissimilarity metrics and bacterial dominance in FFPE samples. Dissimilarity matrices are based on all microbial communities present in paired FF and FFPE CRC tissue microbiome (*y*-axis). In *x*-axes, bacterial dominance is expressed as the sum of the two most abundant orders in FF measured in their matched FFPE samples. Spearman’s correlation coefficient and BH-adjusted *p*-values are indicated on the top of each panel. (**A**–**C**) Sample reads were randomly rarefied to the minimum sample depth (5808 sequences). (**A**,**B**) Sample pairs are ordered based on increasing similarity. Abbreviations: FF; fresh frozen and FFPE; formalin-fixed paraffin-embedded.

Our results showed substantial variability in CRC-associated microbiota across FF-FFPE sample pairs (Figure 5A); thus, we attempted to identify a quality control index for FFPE tissues used in this study.

To address this, the bacterial dominance in FF samples (here defined as the relative abundance of the two most abundant orders) was compared with their matched FFPE sample. Six out of ten sample pairs (129, 134, 119, 123, 135 and 136) were mainly dominated by *Bacteroidales* and *Clostridiales* in both FF and FFPE tissues, using an arbitrary abundance cutoff of 50% (Figure 5B). Moreover, the bacterial dominance in FFPE tissues (defined as the distribution in FFPE tissues of two most abundant orders identified in their matched FF samples) strongly correlated with similarity within each sample pair measured by using different dissimilarity metrics at the ASV level (Bray–Curtis, r = −0.72 and *p* = 0.018; Jaccard, r = −0.72 and *p* = 0.012; Jensen–Shannon, r = −0.74 and *p* = 0.019) (Figure 5C). Concordance between FF and FFPE sample pairs was not significantly influenced by the tumor content (Figure S9). Both compositional and correlation analyses revealed that the four sample pairs (124, 11, 133 and 121) exhibiting higher dissimilarity also showed higher compositional variability and abundance of contaminant taxa (Figure 5A,C). Based on these profiles, sample pairs used in this study were classified as ‘low’ and ‘high’ comparability (Figure S10A). Next, available samples from few other similar studies (published data [10,18]) were classified based on the above-defined criteria (abundance of contaminant taxa and dominance of two most abundant orders within sample pair) (Figure S10B,C) for cross-study comparison. About the published study by Debesa-Tur et al. characterizing CRC-associated microbiota from FFPE tissue types [10], samples were classified according to dominance criteria (if “high”, abundance of two most abundant orders > 50%) and prevalence of typical contaminants in the global composition (Figure S10C). Based on the above-described classification, a comparison between samples virtually having “high” and “low” comparability across the three studies was performed (Figure S11A). Discriminant analysis revealed that “low” comparability samples were enriched in taxa (at both order and genus levels) described as typical contaminants while being virtually absent in the other samples (Figure S11B,C). This would hint at a subset of FFPE tissues having high levels of contaminant bacteria potentially derived from storage or manipulation procedures, in which the microbiota profile would not fully recapitulate that of their matched frozen tissues.

## 4. Discussion

Increasing evidence suggests that the analysis of CRC-associated microbiota can reveal crucial aspects of cancer progression and response to treatments. Given the relatively low similarity between stool and mucosal samples and limited availability of fresh material, FFPE tumor biopsies have a great potential for providing access to a large collection of samples. Nonetheless, their use in high-throughput metagenomic studies has been questioned [14]. In the present study, we profiled the microbiota composition of paired FFPE and FF tissues from a small cohort of CRC patients and found variations in the degree of comparability between preservation methods.

In general, our microbiome profiles were comparable to other available data, in which *Firmicutes, Bacteroidetes* and *Fusobacteria* were reported as the most predominant taxa in CRC samples [41]. Both compositional and diversity analyses revealed distinct microbial communities, suggesting that the microbial composition of FFPE sample types did not completely resemble those of their matched frozen material. One of the most remarkable differences was attributed to the presence of archaeal sequences in FF but not FFPE tissues. It is worth noting that the presence of archaea in the gut microbiome of CRC patients and co-occurring associations with CRC-enriched bacteria, such as *Bacteroides* spp., have been previously reported [42].

Both RNA-ISH and 16S rRNA sequencing were effective at capturing the most prevalent tumor-associated bacteria assessed in this study. Using a 16S rRNA approach, we were able to detect *Fusobacterium*, widely proposed as a diagnostic and prognostic CRC biomarker [43], in most FF samples but only two matched FFPE samples from our cohort. Tumor-associated *Fusobacterium* characterized by 16S rRNA sequencing in FF samples were fully recapitulated by RNA-ISH analysis, with one exception. Conversely, *Fusobacterium* could be detected by 16S on FFPE only in one out of seven RNA-ISH/16S rRNA evaluable pairs, suggesting that 16S sequencing might underestimate *Fusobacterium* abundance in FFPE samples. Using a metagenomics approach, Debesa-Tur et al. were able to find an enrichment of *Fusobacterium* spp. in FFPE colorectal tumor specimens, albeit direct comparison with matched frozen material was not provided [10]. This discrepancy may be linked to the use of optimized extraction and library preparation protocols aimed at improving the analysis of bacterial DNA from FFPE biotypes [10]. *Bacteroides* [44] were also characterized in our study cohort by both 16S rRNA sequencing and RNA-ISH. We were able to visualize *Bacteroides* by RNA-ISH in roughly half of the 16S positive samples. The lower sensitivity shown by RNA-ISH may in part be explained by probe design and/or heterogeneity in the analyzed samples. Importantly, RNA-ISH showed that all *Bacteroides* were tumor-associated, with no or barely detectable bacteria in the adjacent normal mucosa, confirming the putative oncogenic role of this bacteria. An unexpected finding was the ubiquitous detection of *Propionibacterium*, with higher abundance in FFPE samples, as similarly reported in non-tumoral gastric FFPE tissues from another study [18]. *Propionibacterium* spp., such as *P. acnes*, were described as opportunistic pathogens involved in the development of diverse medical conditions, including carcinogenesis [45]. However, *propionibacteria* were also described as contaminants associated to clinical samples and other sources (i.e., laboratory settings or environment), frequently detectable in high-throughput sequencing data [46]. Results from spatial analysis using RNA-ISH in our study indicated that *Propionibacterium* could either act as tumor-associated and/or contaminant bacteria. In contrast to *Fusobacterium* and *Bacteroides* that were significantly enriched in correspondence with the tumor regions with only sporadic presence in adjacent normal mucosa, *Propionibacterium* were randomly distributed across the sample being visualized in both normal and tumor tissue areas as well as in the outer sample margins.

Based on these findings, the RNA-ISH method might be successfully employed in validating and complementing 16S rRNA gene-based microbial profiling, adding valuable spatial information to be considered together with sequencing data for the interpretation of novel potential pathogens or contaminants. Moreover, our results indicate that RNA-ISH may be the preferred method to study selected bacteria in FFPE tissues. This is particularly relevant for *Fusobacterium* due to the emerging role of this bacteria as a putative biomarker, validating the use of this methodology for the extensive characterization of archival FFPE tumor samples from large patient cohorts with associated clinical and outcome data.

Our data also revealed that the microbiota of FF samples was enriched in other bacterial taxa frequently associated with CRC, such as *Collinsella* and *Parvimonas* [47]. Whereas, typical water- and soil-contaminants, including *Paracoccus, Sphingomonas, Pseudomonas* and *Acinetobacter* [48] were increased in FFPE tissues. Although our primary goal was to explore the comparability of CRC-associated microbiota between paired FF and FFPE tissues, this study necessarily addresses technical details regarding contamination issues. Compared with high biomass samples (i.e., stool), contamination is a considerable threat to the accuracy of sequence-based analysis in low biomass samples such as FFPE specimens, blood, or tumor biopsies [48]. Non-sterile conditions during the formalin fixation process may render low biomass samples extremely susceptible to the burden of contaminants, thus obscuring the microbial composition in these sample types [49,50]. Additionally, a high host to bacterial DNA ratio, as found in our samples, potentially derived from the patient, hospital and/or lab personnel can lead to PCR biases, thus reducing amplification efficiency and validity of results [51]. In our study, bacterial taxa documented in the current literature as typical contaminants were identified and then subtracted from the dataset using custom bioinformatic approaches. Nevertheless, the most common strategies for minimizing alterations introduced during FFPE sample processing mainly fall under the remit of wet-lab procedures (i.e., host DNA depletion, microbial enrichment, and DNA repair during the extraction process [50]). Additionally, computational methods such as SourceTracker, and Decontam [52,53] have been developed to identify potential contaminants in high-throughput metagenomic studies and address their in silico removal to improve data accuracy, given that their sensitivity is highly influenced by the number of control samples.

Given the variable impact of FFPE DNA quality in the comparison of our matched-pair cohort, we attempted to implement an internal quality standard to assess the validity of our results. Although we were not able to provide a robust predictive biomarker for FFPE sample quality, our analysis indicated that the predominance of typical contaminants significantly influences the degree of comparability between FF and FFPE samples. Despite the intrinsic limitations and low comparability with FF tissues, the implementation of FFPE tissues in cancer genomics studies may provide new insight into the discovery of novel cancer biomarkers associated with this sample type.

Most of the microbiome studies including tumor samples are limited by small sample sizes [54] and this limitation is also inherent in our study. Although the sample size in our study would be acceptable to gain preliminary insights into the feasibility of using FFPE tissues for characterizing the CRC-associated microbiota, larger sample size is recommended to overcome potential drawbacks derived from the use of FFPE DNA. According to our results, the preservation method did not directly influence the sequencing yield. Nonetheless, sequencing efforts are highly encouraged to obtain sufficient coverage of the bacterial DNA in clinical samples potentially having high levels of human and contaminant sequences.

Besides the presence of contaminants, we were not able to discern additional factors in our study, such as fixation process, time of archiving in formalin, or extraction process [18,55], which may crucially affect the quality of preserved DNA or tumor content. Several studies indicated the paramount importance of using positive and negative controls to assess for potential biases in low biomass studies [37]. Blank controls of the embedding medium were also recommended to assess for potential artifacts introduced during the formalin fixation process [18,20]. Lastly, intrasample heterogeneity might also have an impact on the differences in microbiota composition and further studies addressing this point are needed. Based on these considerations, before a reliable characterization of tumor microbiota is to extend to FFPE samples, then a clear understanding of all potential confounding factors is required to improve downstream analysis and data interpretation. In this context, Walker et al. recently published a comprehensive description of best practice for sequence analysis of bacteria residing in FF and FFPE tumor tissues, providing a guideline for optimized experimental practices and bioinformatic strategies. Furthermore, a number of approaches have been suggested for increasing the potential value of FFPE samples in metagenomics studies, such as the multiplexed 16S rDNA sequencing protocol based on the amplification and computational combination of short regions along the 16S rRNA gene to provide higher resolution of fragmented bacterial DNA [56]. Moreover, frameworks implementing binary encoding, super-resolution imaging and machine learning to analyze the spatial ecology of complex microbial communities at single-cell resolution, such as the HiPR-FISH technique [57] may open up new opportunities for investigations of gut-related alterations, including the role of bacterial biofilms in epithelial barrier alteration and initiation of gastrointestinal tumors.

Considering multiple challenges in inferring tumor-associated microbiota from FFPE tissue samples, these results might be of some external validity to address potential confounding factors.

## 5. Conclusions

Our data show that sample preservation influences the microbiome composition of CRC biopsies, implying that results from frozen might not be directly extrapolated to FFPE, or vice-versa. However, FFPE tissues have the potential to provide a valuable alternative for novel cancer biomarker discovery if appropriate processing conditions and validation are applied. Despite these limitations, 16S sequencing of CRC biopsies bears the potential to recapitulate RNA-ISH profiling and identify potential pathogens involved in CRC development.

## Figures and Tables

**Table 1:** Sequencing statistics per sample group included in this study.

Sequencing Data	FFPE	FF	NTC
# Raw reads	916,957	417,435	67,621
Mean raw reads	83,359	46,381	
# Filtered reads	492,972	359,237	57,201
Mean filtered reads	44,815	39,915	
# Final high-quality reads	181,046	142,325	24,537
Mean final high-quality reads	16,458	15,814	
% Chimeric/Raw reads	79%	67%	64%
# Number ASVs	389	493	

Abbreviations: FF; fresh frozen, FFPE; formalin-fixed paraffin-embedded, NTC; no-template control, ASVs; amplicon sequence variants and #; number.

**Table 2:** Presence or absence (+/−) of bacterial genera characterized by RNA-ISH and 16S rRNA sequencing for each of the sample pairs tested evaluable for RNA-ISH.

	*Bacteroides*			*Fusobacterium*			*Propionibacterium*		
ID	RNA-ISH (FFPE)	16S (FFPE)	16S (FF)	RNA-ISH (FFPE)	16S (FFPE)	16S (FF)	RNA-ISH (FFPE)	16S (FFPE)	16S (FF)
119	−	+	+	+	−	−	+	+	+
121	−	+	+	−	−	−	+	+	+
123	+	+	+	+	+	+	+	+	+
124	−	−	+	+	−	+	+	+	+
129	+	+	+	+	−	+	+	+	+
135	−	+	+	−	−	−	+	+	+
136	+	+	+	+	−	+	+	+	−

Abbreviations: FF; fresh frozen and FFPE; formalin-fixed paraffin-embedded.

## Data Availability

Raw data from this study are available in the EBI Short Read Archive under the study accession number PRJEB46353.

## Supplementary Materials

The following are available online at https://www.mdpi.com/article/10.3390/cancers13215421/s1, Figure S1. PCR amplification profiles of paraffin controls. Figure S2. 16S rRNA gene sequencing yield. Figure S3. Comparison of NTC-associated ASVs between FF and FFPE groups. Figure S4. Archae content in FF and FFPE tissue samples. Figure S5. Microbial composition at the order level. Figure S6. Fusobacterium, Bacteroides and Propionibacterium in FF and FFPE sample pairs. Figure S7. Fusobacterium putative species identified in FF and FFPE samples. Figure S8. Fusobacterium, Bacteroides and Propionibacterium in normal and colorectal cancer FFPE tissues. Figure S9. Correlation between microbiome concordance and tumor content in FF and FFPE sample pairs. Figure S10. Comparison of bacterial composition across studies. Figure S11. Microbiota-associated profiles in FFPE tissue samples with “high” and “low” comparability. Table S1. Pathologic data of the samples’ patients of the study. Table S2. Summary of the bacterial detection by RNA-ISH in FFPE samples and 16S on FF and FFPE samples.

## Abbreviations

16S rRNA	16S ribosomal RNA
ASV	Amplicon sequence variant
CRC	Colorectal cancer
DNA	Deoxyribonucleic acid
FF	Fresh frozen
FFPE	Formalin-fixed paraffin-embedded
HiPR-FISH	High Phylogenetic Resolution microbiome mapping by Fluorescence In-Situ Hybridization
RNA-ISH	RNA in situ hybridization
NCBI	National Center for Biotechnology Information
NGS	Next-generation sequencing
NTC	Negative template control
PCoA	Principal Coordinates Analysis
PCR	Polymerase chain reaction
