# Supplementary material for: Performance of 16S Metagenomic Profiling in Formalin-Fixed Paraffin-Embedded versus Fresh-Frozen Colorectal Cancer Tissues
Source: Cancers (Basel). 2021 Oct 29;13(21):5421. doi: 10.3390/cancers13215421 (PMC8582506; doi:10.3390/cancers13215421)
Supplement: Supplementary file 1 [file cancers-13-05421-s001.zip › cancers-1403902-supplementary.pdf]

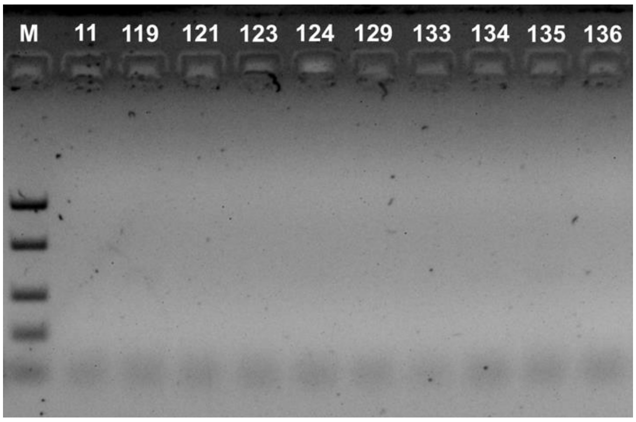

Figure S1. PCR amplification profiles of paraffin controls. (A) Gel electrophoresis of V3-V4 16S rRNA gene amplicon in paraffin controls. DNA ladder is marked as 'M' and numbers indicates the corresponding blank paraffin block.

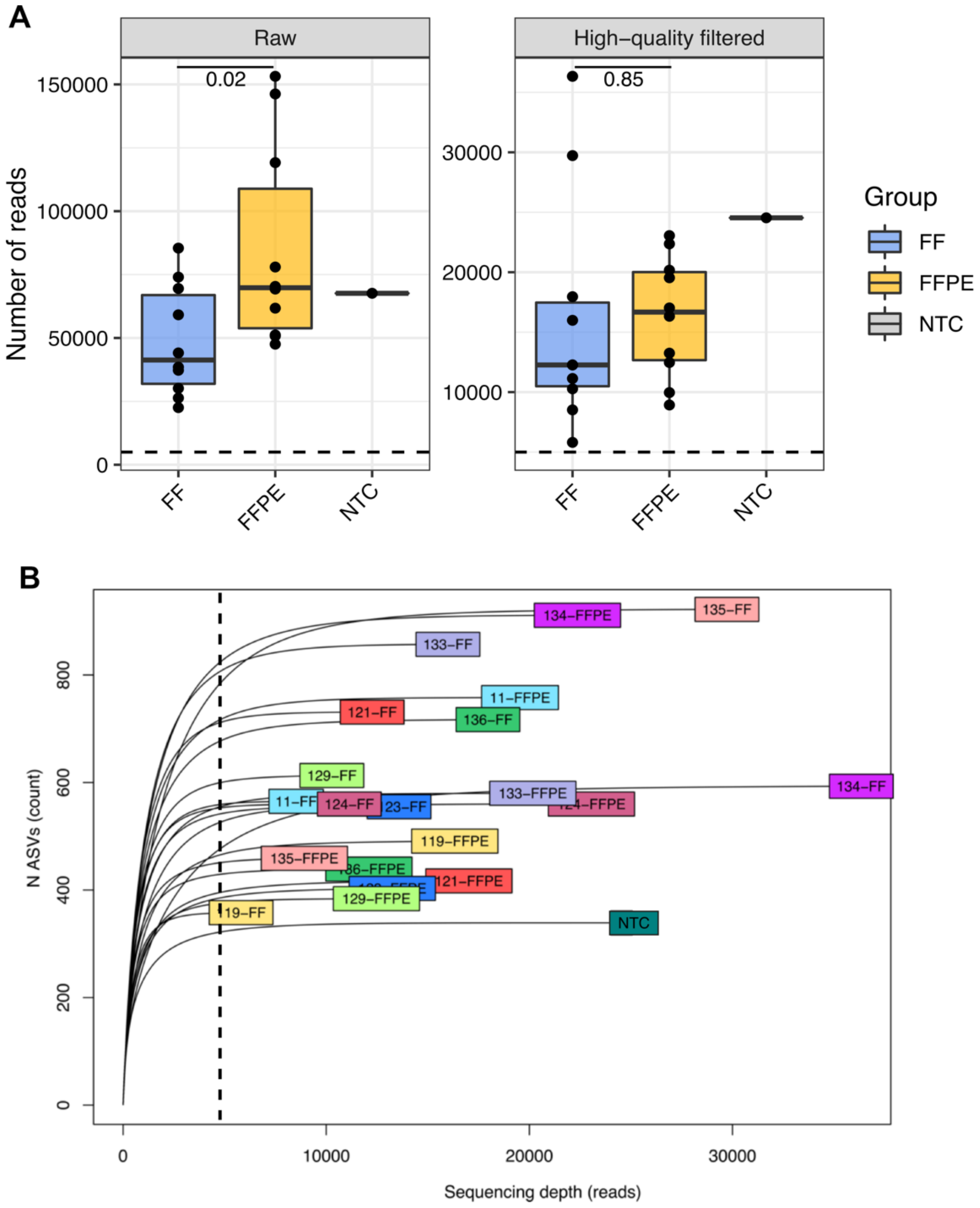

Figure S2. 16S rRNA gene sequencing yield. (A) Boxplots displaying raw and chimera-removed, high-quality filtered reads in FF, FFPE and negative template control samples. The horizontal dashed line indicates 5,000 read number threshold (B) Rarefaction curve plot of all 21 samples (one curve per sample) showing number of ASVs as a function of the read number. The vertical dashed line indicates 5,000 read number threshold Curves reached asymptote at the cut-off of 5000 reads, excepted for sample 124-FF. Abbreviations: FF; fresh frozen, FFPE; formalin-fixed paraffin-embedded and NTC; no-template control.

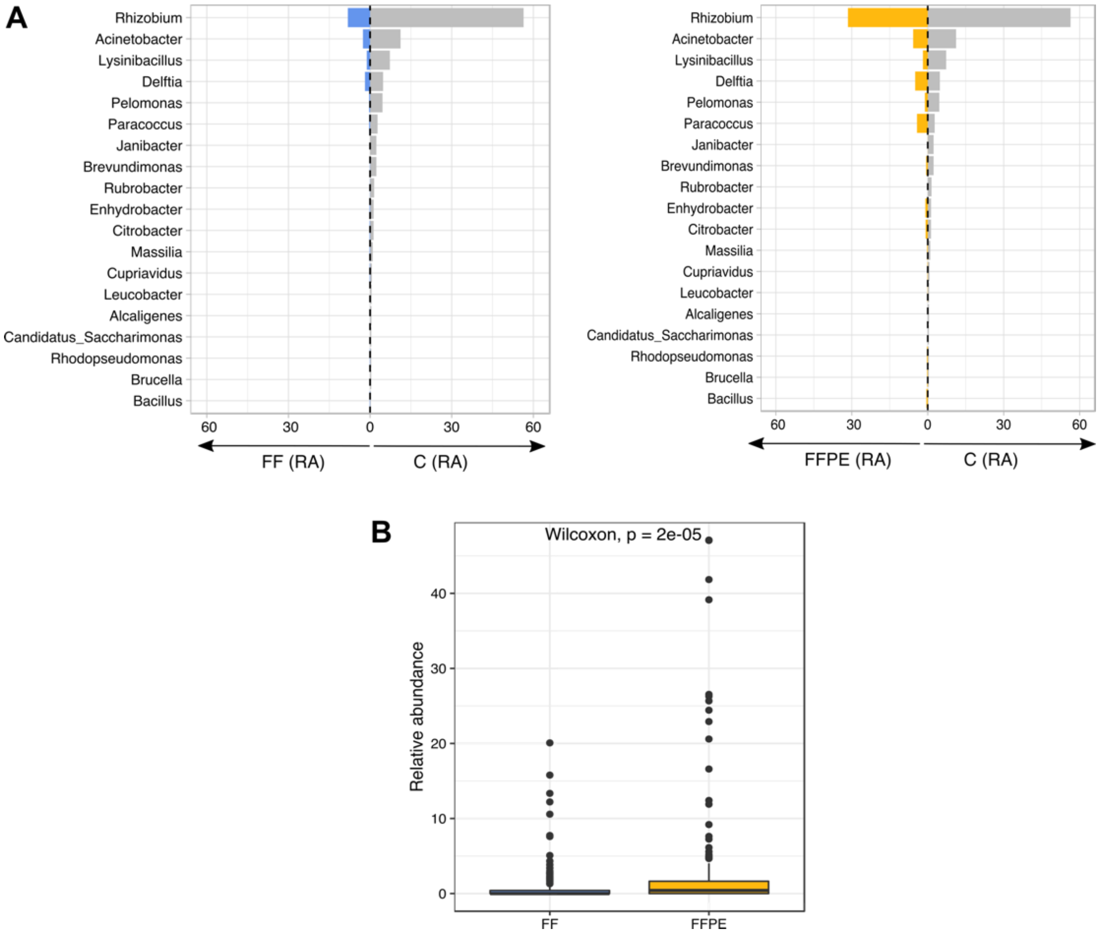

Figure S3. Comparison of negative control-associated ASVs between FF and FFPE groups. (A) Horizontal bars showing the relative abundance (as percentage) of bacterial genera identified in the negative template control (grey) and corresponding distribution in FF (blue) and FFPE (yellow) samples. Bars represent mean value per taxa of each group. (B) Comparison of control-associated ASVs between FF and FFPE methods. Dots indicates outliers of control-associated ASVs at the genus level.

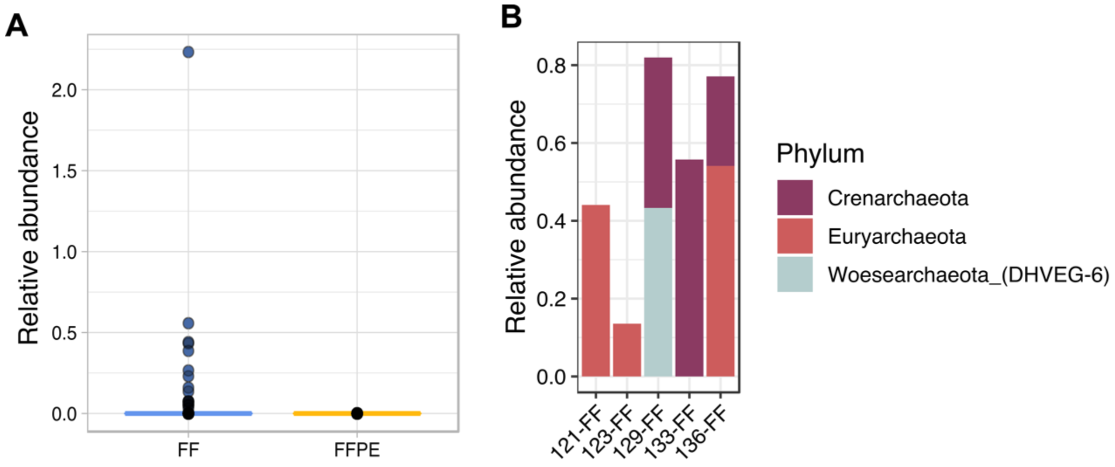

Figure S4. Archaea content in FF and FFPE tissue samples. (A) Box plots showing the relative abundance of ASVs corresponding to archaeal sequences between FF and FFPE tissue samples. Each dot corresponds to a single ASV. Archaeal sequences were identified exclusively in FF tissue samples. (B) Bar plot displaying the relative abundance of archaeal phyla in FF tissue samples.

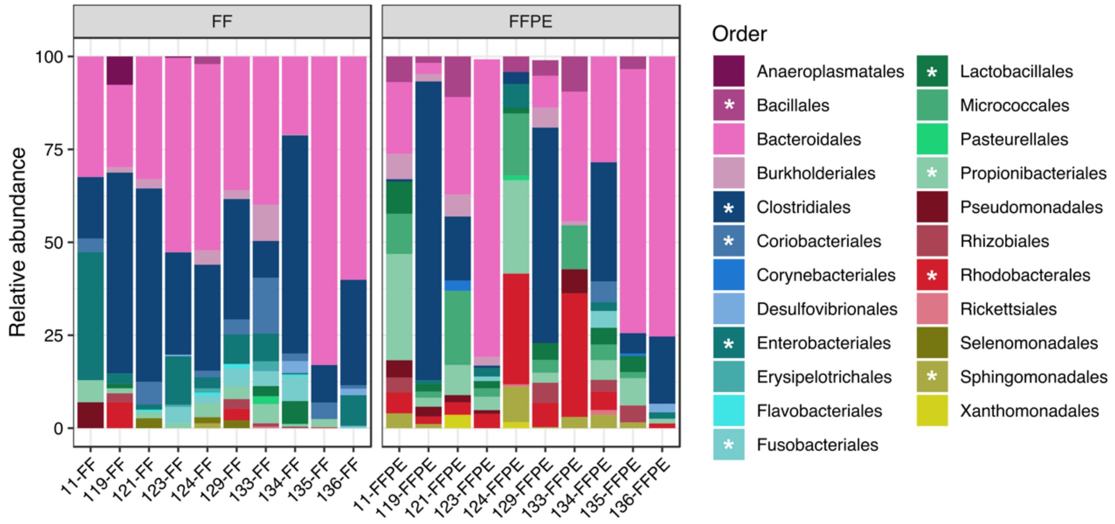

Figure S5. Microbial composition at the order level. Stacked bar charts illustrating the relative abundance (in percentage) of bacterial orders found in each sample pair. Each vertical bar represents one sample. All bacterial orders are represented by a colour shown in the figure key. Significantly different phyla are indicated by a white asterisk ($p \leq 0.05$, Wilcoxon paired test).

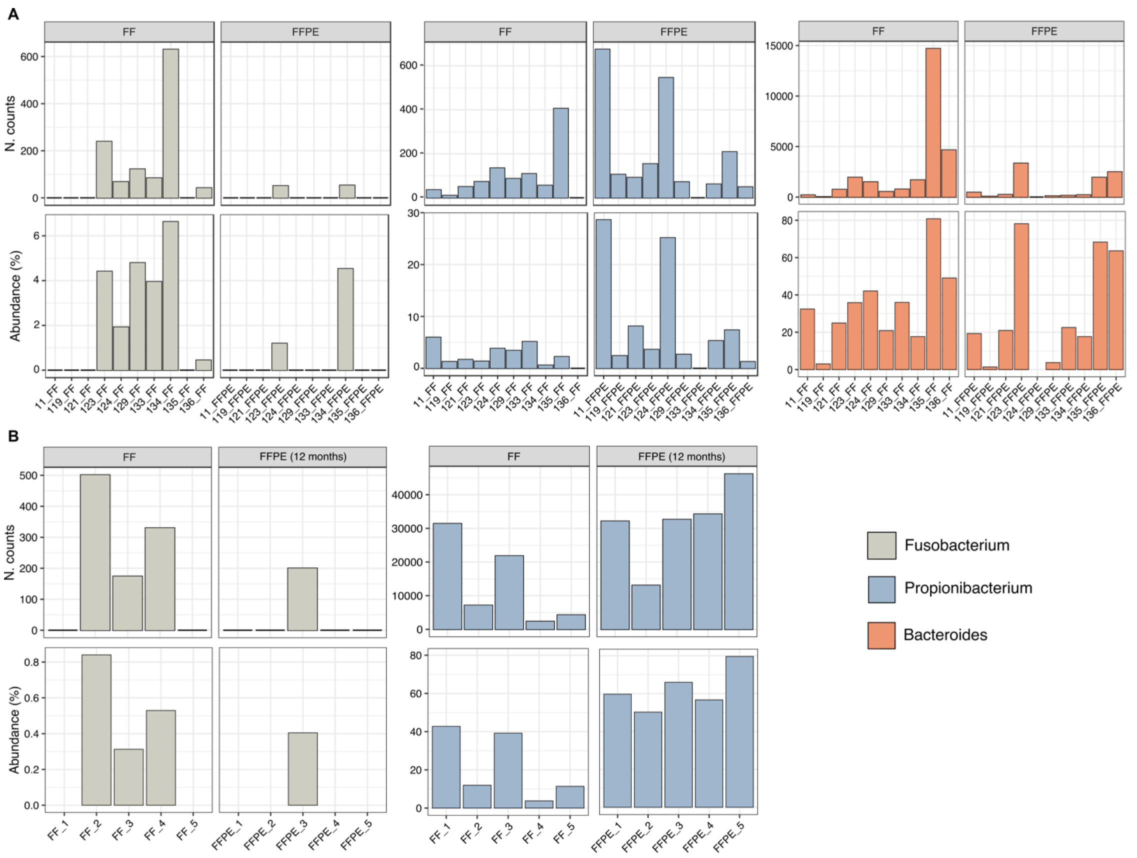

Figure S6. ***Fusobacterium*, *Bacteroides* and *Propionibacterium* in FF and FFPE sample pairs.** Bar plots showing count number and relative abundance (in percentage) of selected genera in FF and FFPE (A) colorectal cancer tissues from the current study and (B) non-tumoral gastric tissues from published 16S rRNA-seq data (Pinto-Ribeiro et al., PRJNA721607).

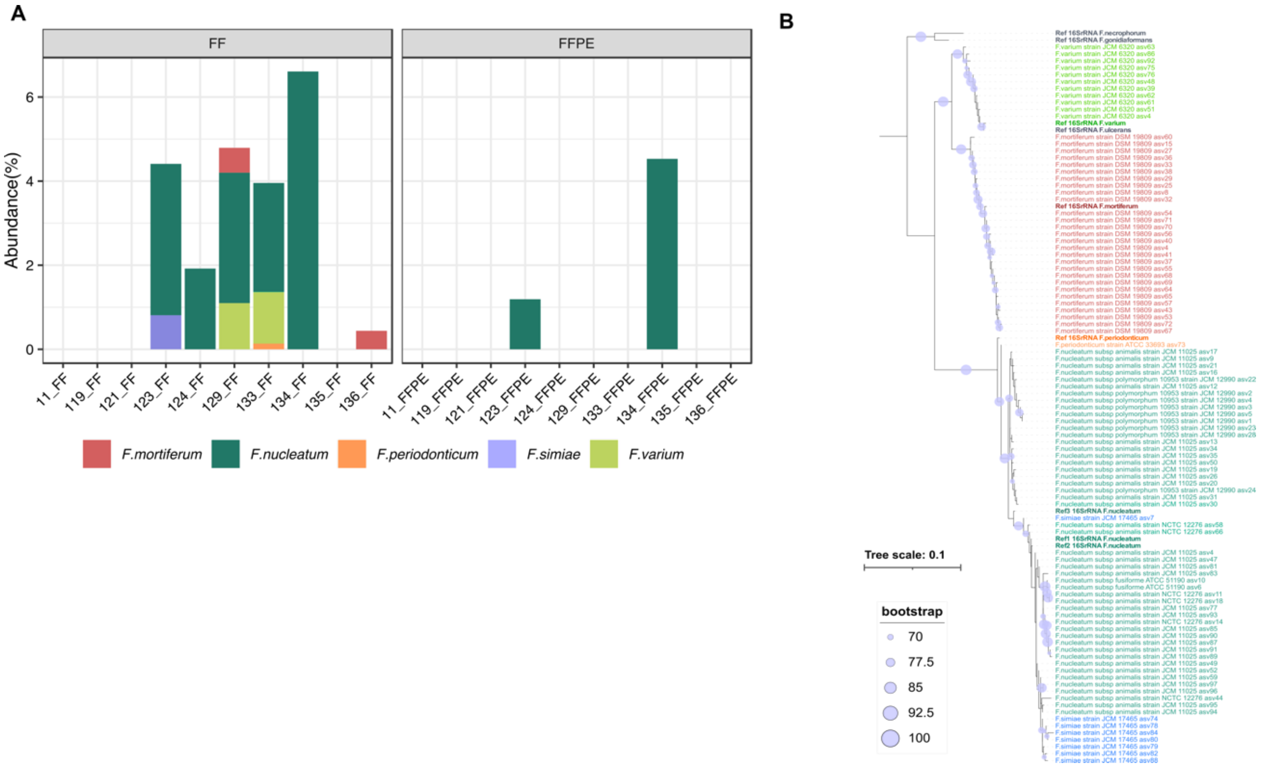

Figure S7. *Fusobacterium* putative species identified in FF and FFPE samples. (A) Bar plot displaying relative abundances of *Fusobacterium*-assigned putative species across the samples. (B) Phylogenetic tree showing the genetic relationships among *Fusobacterium*-associated ASVs and reference 16S gene of *Fusobacterium* spp. 16S gene reference sequences downloaded from PATRIC database were indicated in bold. *Fusobacterium necrophorum* and *Fusobacterium gonidiaformans* were used as an outlying group. The size of blue circles represents the bootstrap value based on 1000 replications. Fusobacterial species are color-coded according to classification in (A).

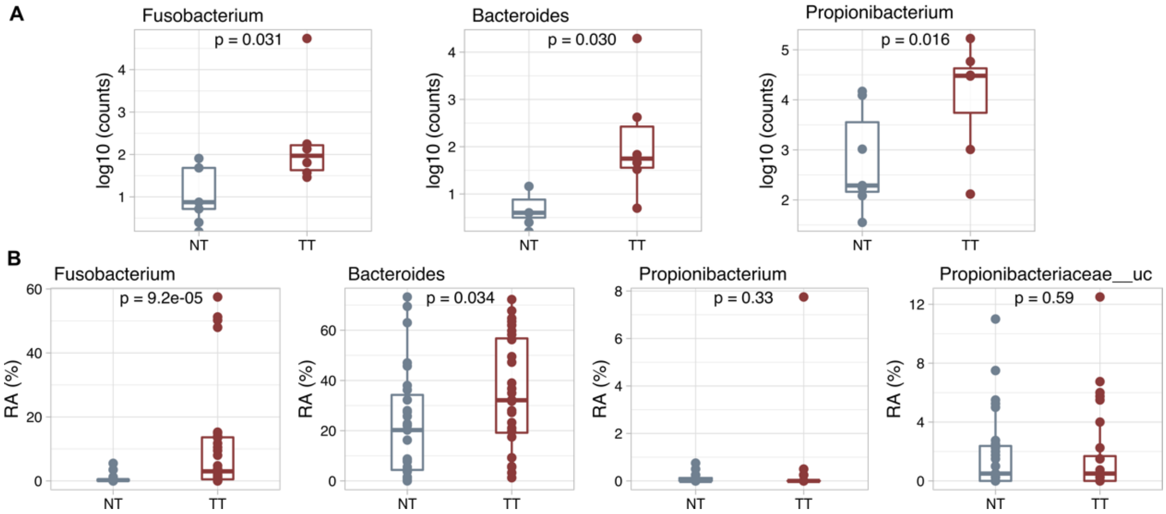

Figure S8. *Fusobacterium, Bacteroides* and *Propionibacterium* in normal and colorectal cancer FFPE tissues. Boxplots of Wilcoxon Signed Rank tests for selected genera profiled (A) in the current study by RNA-ISH (log10-transformed counts) and (B) in published patient metagenomic data (Debesa-Tur et al., PRJEB34333) (relative abundance as percentage). Abbreviations: NT, normal tissue; TT, tumoral tissue.

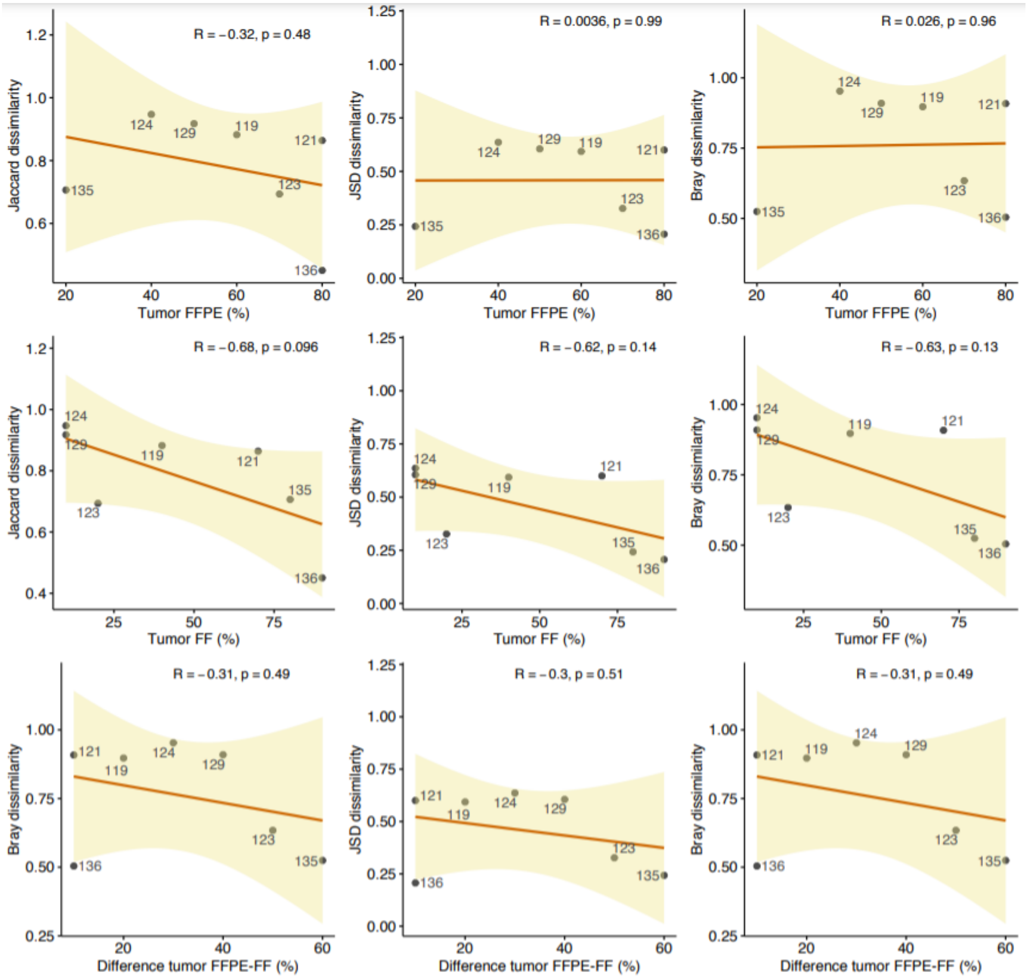

Figure S9. Correlation between microbiome concordance and tumor content in FF and FFPE sample pairs. Scatter plots showing spearman's correlation between Bray-Curtis, Jaccard and Jensen-Shannon dissimilarity metrics and quantification of tumor percentage in FFPE and FF samples. Dissimilarity matrices are based on all microbial communities present in paired FF and FFPE CRC tissue microbiome (y-axis). Spearman's correlation coefficient and BH-adjusted p-values are indicated on the top of each panel. For dissimilarity matrix measurements, sample reads were randomly rarefied to the minimum sample depth (5808 sequences).

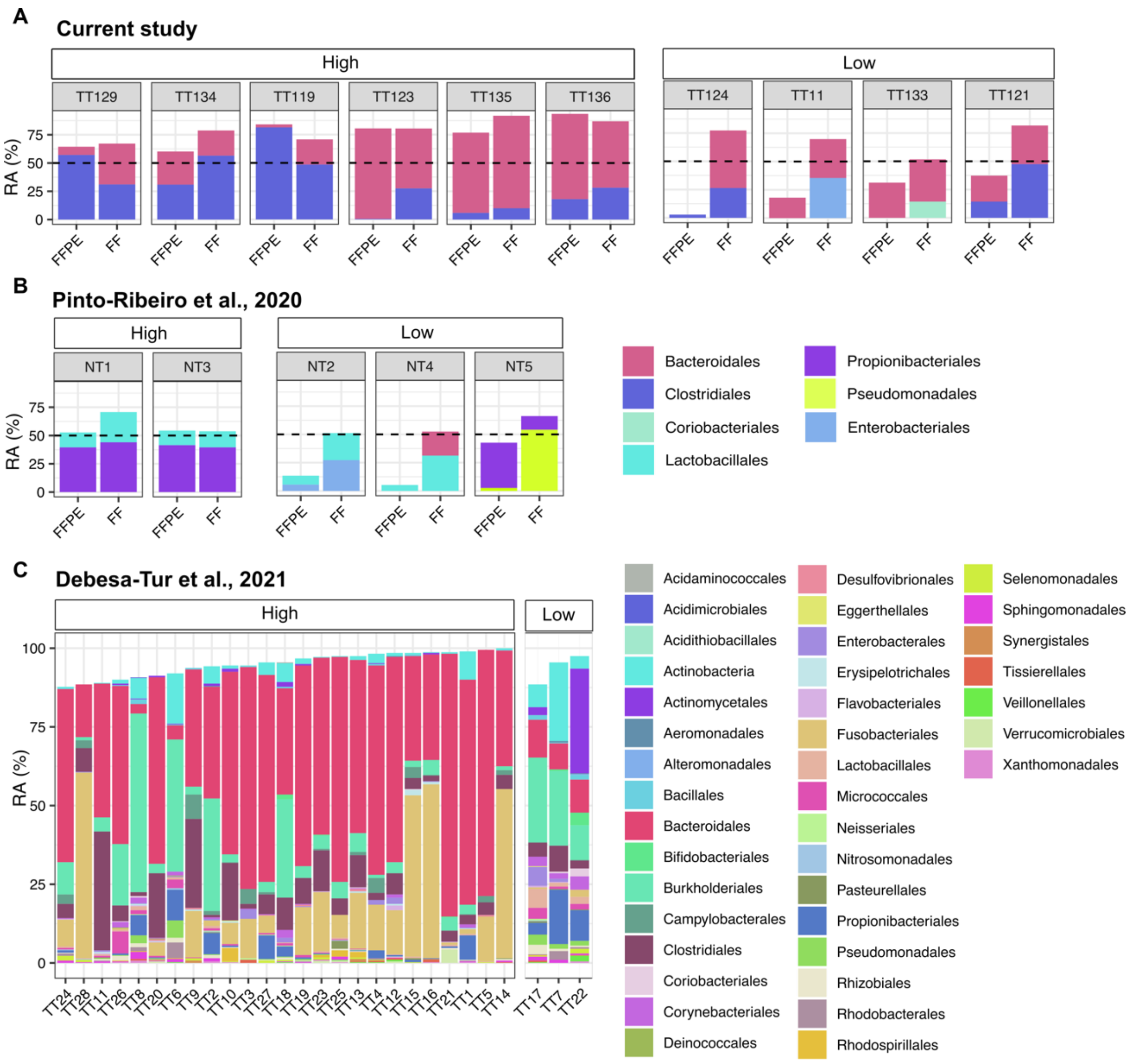

Figure S10. Comparison of bacterial composition across studies. Relative abundance (percentage) of two most abundant bacterial orders in FFPE stored A) colorectal cancer tissues (current study) and B) normal gastric mucosa (published data Pinto-Ribeiro et al., PRJNA721607). C) Bacterial order abundance in FFPE colorectal cancer tissues in published patient metagenomic data (Debesa-Tur et al., PRJEB34333). (A-C) Sample classification as "high" and "low" comparability was based on bacterial dominance and prevalence of typical contaminant taxa.

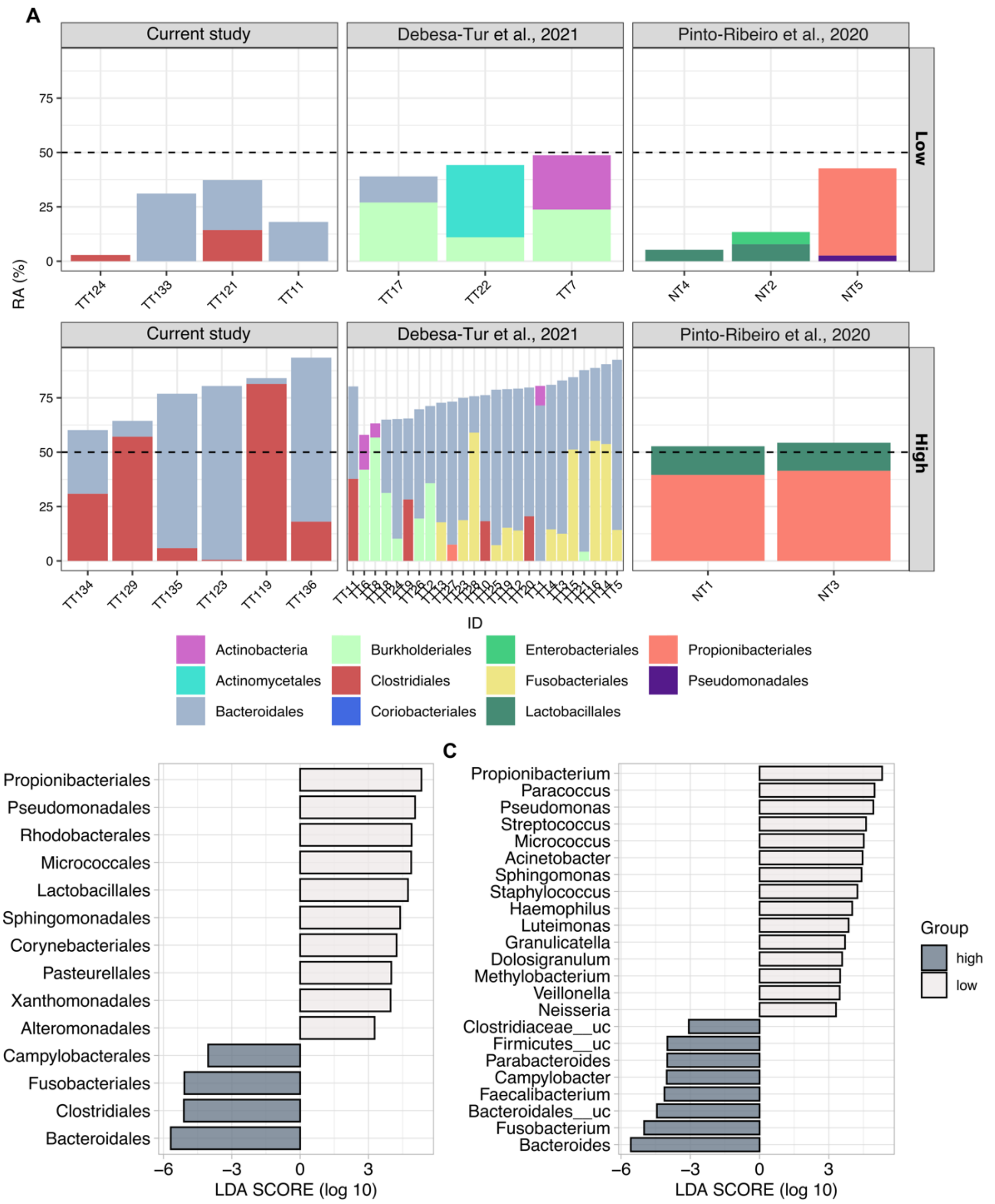

Figure S11. Microbiota-associated profiles in FFPE tissue samples with "high" and "low" comparability. (A) Distribution of two most abundant bacterial orders in FFPE tissue samples across the current study and published datasets (Pinto-Ribeiro et al., 2020; PRJNA721607 and Debesa-Tur et al., 2021; PRJEB34333). Discriminant taxa at the (B) order and (C) genus levels between FFPE tissue microbiome classified as "low" and "high" comparability. Samples within each study were grouped together for LEfSe analysis.

Table S1. Pathologic data of the tumor samples included in the study.

Patient ID	HISTOLOGY	GRADE	pT	pN	pM	STAGE	TUMOR PERCENTAGE FFPE	TUMOR PERCENTAGE FF
11	ADENOCARCINOMA	2	4a	N2b	M1	IV	50%	60%
119	ADENOCARCINOMA	2	3	0	x	II	60%	40%
121	ADENOCARCINOMA	2	3	0	x	II	80%	70%
123	ADENOCARCINOMA	2	3	0	x	II	70%	20%
124	ADENOCARCINOMA	3	3	0	x	II	40%	10%
129	ADENOCARCINOMA	2	3	0	x	II	50%	10%
133	ADENOCARCINOMA	2	3	0	x	II	20%	90%
134	ADENOCARCINOMA	3	3	0	x	II	30%	Na
135	ADENOCARCINOMA	2	3	0	x	II	20%	80%
136	ADENOCARCINOMA	2	4a	0	x	II	80%	90%

Na, not available.

Table S2. Summary of the bacterial detection by RNA-ISH in FFPE samples and 16S on FF and FFPE samples.

TARGET	Sample ID	QC ISH	COUNT	ISH RESULT	TOTAL COUNT BACTERIA FF	TOTAL COUNT BACTERIA FFPE
Bacteroides	11	NO PASS	3	NA	196	456
Bacteroides	119	PASS	46	NEG	24	61
Bacteroides	121	PASS	33	NEG	745	238
Bacteroides	123	PASS	19490	POS	1945	3340
Bacteroides	124	PASS	12	NEG	1490	0
Bacteroides	129	PASS	420	POS	532	101
Bacteroides	133	NO PASS	59	NA	766	143
Bacteroides	134	NO PASS	68	NA	1683	207
Bacteroides	135	PASS	5	NEG	14691	1943
Bacteroides	136	PASS	346	POS	4652	2486
EB16S	11	NO PASS	2	NA	604	2360
EB16S	134	NO PASS	30	NA	9506	1170
EB16S	133	NO PASS	60	NA	2127	634
EB16S	124	PASS	1175	POS	3538	2172
EB16S	121	PASS	17877	POS	121	1136
EB16S	129	PASS	33495	POS	2546	2698
EB16S	136	PASS	129835	POS	18175	2842
EB16S	123	PASS	174253	POS	5421	4271
EB16S	135	PASS	147281	POS	18175	2842
EB16S	119	PASS	1292	POS	788	4405
Fusobacterium	11	NO PASS	0	NA	0	0
Fusobacterium	133	NO PASS	12	NA	84	0
Fusobacterium	134	NO PASS	29	NA	630	53
Fusobacterium	121	PASS	64	NEG	0	0
Fusobacterium	135	PASS	37	NEG	0	0
Fusobacterium	119	PASS	135	POS	0	0
Fusobacterium	136	PASS	287	POS	42	0
Fusobacterium	129	PASS	177	POS	122	0
Fusobacterium	124	PASS	338	POS	68	0
Fusobacterium	123	PASS	54412	POS	239	51
Propionibacterium	11	NO PASS	46	NA	36	675
Propionibacterium	134	NO PASS	131	NA	56	62
Propionibacterium	133	NO PASS	217	NA	109	0
Propionibacterium	135	PASS	1018	POS	406	209
Propionibacterium	124	PASS	1995	POS	135	546
Propionibacterium	129	PASS	31076	POS	87	72
Propionibacterium	121	PASS	30279	POS	50	92

Propionibacterium	119	PASS	86597	POS	10	106
Propionibacterium	123	PASS	168543	POS	73	154
Propionibacterium	136	PASS	230236	POS	0	49
